# Social distancing and epidemic resurgence in agent-based susceptible-infectious-recovered models

**DOI:** 10.1038/s41598-020-80162-y

**Published:** 2021-01-08

**Authors:** Ruslan I. Mukhamadiarov, Shengfeng Deng, Shannon R. Serrao, Priyanka, Riya Nandi, Louie Hong Yao, Uwe C. Täuber

**Affiliations:** 1 Department of Physics and Center for Soft Matter and Biological Physics, Virginia Tech, Blacksburg, VA 24061 USA; 2 Key Laboratory of Quark and Lepton Physics (MOE) and Institute of Particle Physics, Central China Normal University, Wuhan, 430079 China; 3 Faculty of Health Sciences, Virginia Tech, Blacksburg, VA 24061 USA

**Keywords:** Biophysics, Diseases, Physics

## Abstract

Once an epidemic outbreak has been effectively contained through non-pharmaceutical interventions, a safe protocol is required for the subsequent release of social distancing restrictions to prevent a disastrous resurgence of the infection. We report individual-based numerical simulations of stochastic susceptible-infectious-recovered model variants on four distinct spatially organized lattice and network architectures wherein contact and mobility constraints are implemented. We robustly find that the intensity and spatial spread of the epidemic recurrence wave can be limited to a manageable extent provided release of these restrictions is delayed sufficiently (for a duration of at least thrice the time until the peak of the unmitigated outbreak) and long-distance connections are maintained on a low level (limited to less than five percent of the overall connectivity).

## Introduction

The COVID-19 pandemic constitutes a severe global health crisis. Many countries have implemented stringent non-pharmaceutical control measures that involve behavioral change of the public such as social distancing, using face-coverings and mobility reduction enforced by lockdowns in their populations. This has led to remarkably successful deceleration and significant ‘flattening of the curve’ of the infection outbreaks, albeit at tremendous economic and financial costs^1,2^. At this point, societies are in dire need of designing a secure (partial) exit strategy wherein the inevitable recurrence of the infection among the significant non-immune fraction of the population can be thoroughly monitored with sufficient spatial resolution and reliable statistics, provided that dependable, frequent, and widespread virus testing capabilities are accessible and implemented. Until an effective and safe vaccine is widely available, this would ideally allow the localized implementation of rigorous targeted disease control mechanisms that demonstrably protect people’s health while the paralyzed branches of the economy are slowly rebooted.

Mathematical analysis and numerical simulations of infection spreading in generic epidemic models are crucial for testing the efficacy of proposed mitigation measures, and the timing and pace of their gradual secure removal. Specifically, the employed mathematical models need to be (i) stochastic in nature in order to adequately account for randomly occurring or enforced disease extinction in small isolated communities, as well as for rare catastrophic infection boosts and (ii) spatially resolved such that they properly capture the significant emerging correlations among the susceptible and immune subpopulations. These distinguishing features are notably complementary to the more detailed and comprehensive computer models utilized by researchers at the University of Washington, Imperial College London, the Virginia Bioinformatics Institute, and others: see, e.g.,^3–8^.

We report a series of detailed individual-based Monte Carlo computer simulation studies for stochastic variants^9,10^ of the paradigmatic Susceptible-Infectious-Recovered (*SIR*) model^11,12^ for a community of about 100,000 individuals. To determine the robustness of our results and compare the influence of different contact characteristics, we ran our stochastic model on four distinct spatially structured architectures, namely i) regular two-dimensional square lattices, wherein individuals move slowly and with limited range, i.e., spread diffusively; ii) two-dimensional small-world networks that in addition incorporate substantial long-distance interactions and contaminations; and finally on iii) random as well as iv) scale-free social contact networks. For each setup, we investigated epidemic outbreaks with model parameters informed by the known COVID-19 data^6,13^. To allow for a direct comparison, we extracted the corresponding effective infection and recovery rates by fitting the peak height and the half-peak width of the infection growth curves with the associated classical deterministic SIR rate equations that pertain to a well-mixed setting. We designed appropriate implementations of social distancing and contact reduction measures on each architecture by limiting or removing connections between individuals. This approach allowed us to generically assess the efficacy of non-pharmaceutical control measures.

Although each architecture entails varied implementations of social distancing measures, we find that they all robustly reproduce both the resulting reduced outbreak intensity and growth speed. As anticipated, a dramatic resurgence of the epidemic occurs when mobility and contact restrictions are released too early. Yet if stringent and sufficiently long-lasting social distancing measures are imposed, the disease may go extinct in the majority of isolated small population groups. In our spatially extended lattice systems, disease spreading then becomes confined to the perimeters of a few larger outbreak regions, where it can be effectively localized and specifically targeted. For the small-network architecture, it is however imperative that all long-range connections remain curtailed to a very low percentage for the control measures to remain effective. Intriguingly, we observe that an infection outbreak spreading through a static scale-free network effectively randomizes its connectivity for the remaining susceptible nodes, whence the second wave encounters a very different structure.

In the following sections, we briefly describe the methodology and algorithmic implementations as well as pertinent simulation results for each spatial or network structure; additional details are provided in the Supplementary Materials. We conclude with a comparison of our findings and a summary of their implications.

## Results

### Square lattices with diffusive spreading

Our first architecture is a regular two-dimensional square lattice with linear extension $L=448$ subject to periodic boundary conditions (i.e., on a torus). Initially, $N = S\left(0\right)+ I\left(0\right)+ R\left(0\right)=\mathrm{100,000}$ individuals with fixed density $\rho =N/{L}^{2}\approx 0.5$ are randomly placed on the lattice, with at most one individual allowed on each site. Almost the entire population begins in the susceptible state $S\left(0\right)$; we start with only 0.1% infected individuals, $I\left(0\right)=100$, and no recovered (immune) ones, $R\left(0\right)=0$. We note that in stochastic simulations, random fluctuations often lead to the initial infectious population recovering so fast that the epidemic dies out before it can cause an outbreak; therefore we chose to seed the system with 100 randomly placed infected individuals. This initial configuration is moreover motivated by enforced lockdowns and travel restrictions which essentially stops the external influx of new infections. Subsequently, all individuals may move to neighboring empty lattice sites with diffusion rate $d$ (here we set this hopping probability to $1$). Upon their encounter, infectious individuals irreversibly change the state of neighboring susceptible ones with set rate $r$: $S+I \to I+I$. Any infected individual spontaneously recovers to an immune state with fixed rate $a$: $I \to R$. (Details of the simulation algorithm are presented in the Supplementary Materials.) For the recovery period, we choose $1/a\cong$ 6.667 days (1 day is equivalent to one Monte Carlo step, *MCS*) informed by known COVID-19 characteristics^13^. To determine the infection rate $r$, we run simulations for various values, fit the peak height and width of the ensuing epidemic curves with the corresponding SIR rate equations to extract the associated basic reproduction ratio ${R}_{0}$ (as explained in the Supplementary Materials, see Figure S1), and finally select that value for $r$ for our individual-based Monte Carlo simulations that reproduces the ${R}_{0}\approx 2.4$ for COVID-19^6^. We perform $100$ independent simulation runs with these reaction rates, from which we obtain the averaged time tracks for $I(t)$ and $R(t)$, while of course $S\left(t\right)=N-I\left(t\right)-R(t)$ and $R\left(t\right)= a{\int }_{0}^{t}I\left(t{^{\prime}}\right) dt{^{\prime}}$.

The standard classical SIR deterministic rate equations assume a well-mixed population and constitute a mean-field type of approximation wherein stochastic fluctuations and spatial as well as temporal correlations are neglected; see, e.g.,^14,15^*.* Near the peak of the epidemic outbreak, when many individuals are infected, this description is usually adequate, albeit with coarse-grained `renormalized’ rate parameters that effectively incorporate fluctuation effects at short time and small length scales. However, the mean-field rate equations are qualitatively insufficient when the infectious fraction $I(t)/N$ is small, whence both random number fluctuations and the underlying discreteness and associated internal demographic noise become crucial^14–16^. Already near the epidemic threshold, which constitutes a continuous dynamical phase transition far from thermal equilibrium, c.f. Figure S2 in the Supplementary Materials, the dynamics is dominated by strong critical point fluctuations. These are reflected in characteristic initial power laws rather than simple exponential growth of the $I(t)$ and $R(t)$ curves^17^, as demonstrated in Figure S1 (Supplemental Information).

Nor can the deterministic rate equations capture stochastic disease extinction events that may occur at random in regions where the infectious concentration has reached small values locally. The rate equations may be understood to pertain to a static and fully connected network; in contrast, the spreading dynamics on a spatial setting continually rewires any infectious links keeping the epidemic active^8,18^. Consequently, once the epidemic outbreak threshold is exceeded, the *SIR* rate equations markedly underestimate the time-integrated outbreak extent reflected in the ultimate saturation level ${R}_{\infty }=R\left(t\to \infty \right)$, as is apparent in the comparison Figure S1 (Supplemental Information).

Once the instantaneous infectious fraction of the population has reached the threshold 10%, $I\left(t\right)=0.1 N$, we initiate stringent social distancing that we implement through a strong repulsive interaction between any occupied lattice sites (with ${n}_{i}=1$), irrespective of their states $S,$ $I$, or $R$; and correspondingly an attractive force between filled and empty (${n}_{i}=0$) sites, namely the repulsive interaction energy ${V(\{n}_{i}\})=K {\sum }_{<i,j>}\left(2 {n}_{i}-1\right) \left(2 {n}_{j}-1\right)$ with dimensionless strength $K=1$, where the sum extends only over nearest-neighbor pairs on the square lattice. The transfer of any individual from an occupied to an adjacent empty site is subsequently determined through the ensuing energy change $\Delta V$ by the Metropolis transition probability $w= \min \{1, exp(-\Delta V)\}$^19,20^, which replaces the unmitigated hopping rate $d$. As a result, both the mobility as well as any direct contact between individuals on the lattice are quickly and drastically reduced. With this social distancing mechanism, our system effectively operates like an adaptive network^21^, where all types of links, rather than only the *S*-*I* links^22^, tend to be dynamically suppressed during the lockdown period. For sufficiently small total density $\rho = N/{L}^{2}$, most of the individuals eventually become completely isolated from each other. For our $\rho =0.5$, the disease will continue to spread for a short period, until the repulsive potential has induced sufficient spatial anti-correlations between the susceptible individuals. The social-distancing interaction is sustained for a time duration $T$, and then switched off again.

**Figure 1:**
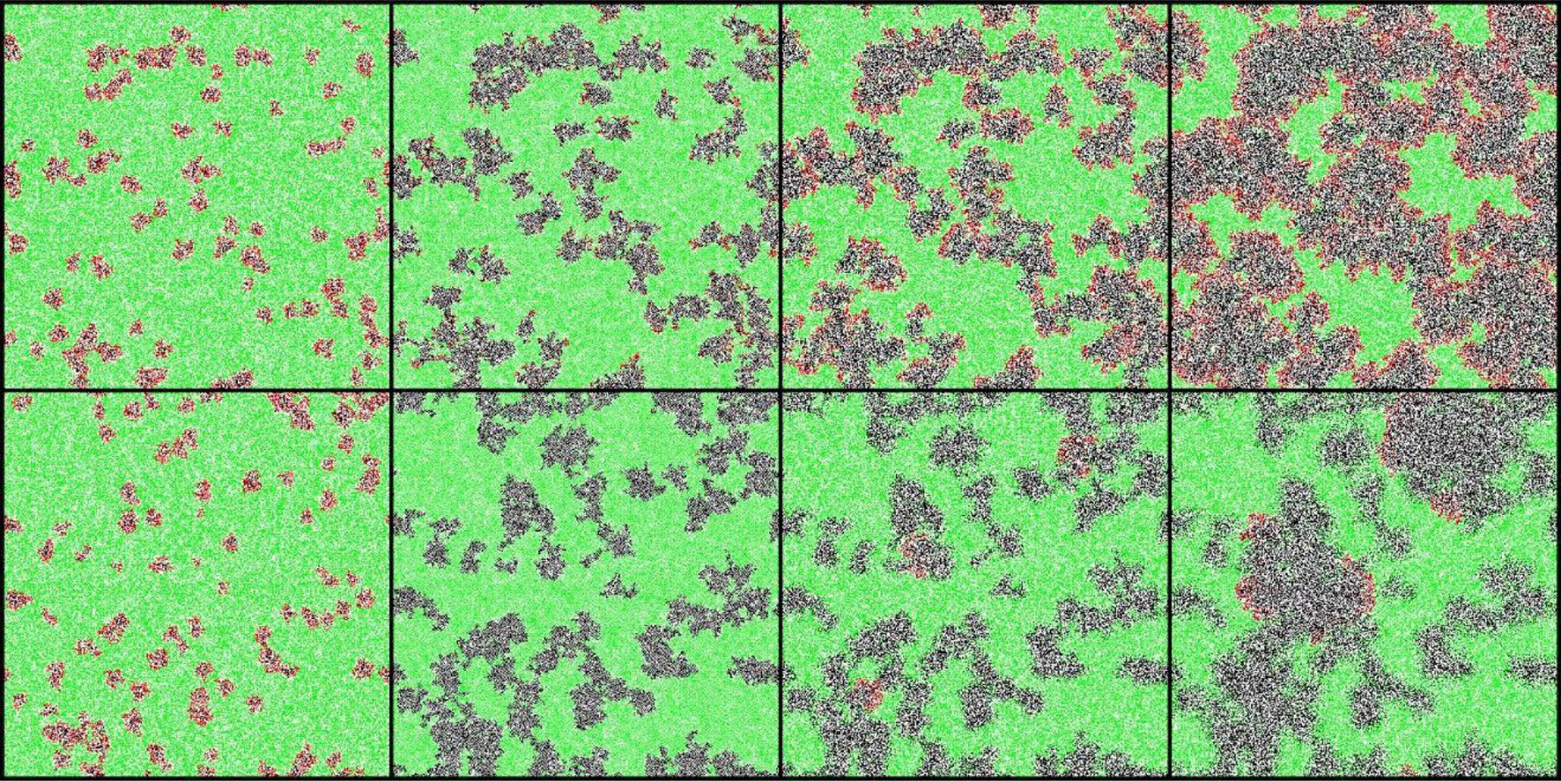
Stochastic *SIR* model simulation snapshots on a square lattice (with periodic boundary conditions). `Social distancing’ is turned on when the number of infective individuals reaches $I(t)= 0.1 N,$ and subsequently maintained for a duration $T=22 MCS=2/a$ in the top, and $T=110 MCS=10/a$ in the bottom row. The green color marker is used for susceptible individuals, while red indicates infected and black recovered (immune or deceased) individuals. The first snapshots (leftmost column) capture the instance when mitigation is implemented. The second column marks the time when social distancing is turned off after additional time $T$ has elapsed. The third and fourth columns show the ensuing spread of the disease. With extended social distancing duration $T$ (bottom row), the infection becomes more likely to be driven to extinction in confined contact regions. Hence the number of active outbreak centers decreases drastically, which could facilitate disease control through effective testing and tracking.

**Figure 2:**
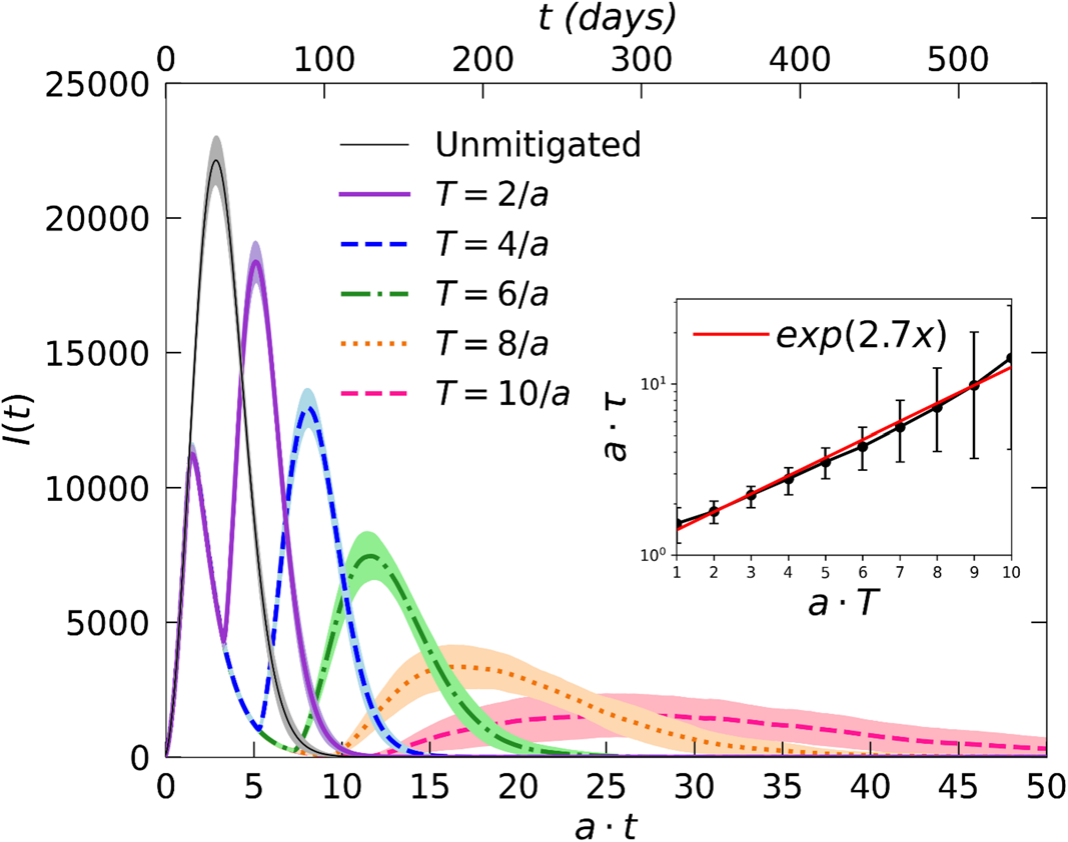
Infection curves $I\left(t\right)$ for the stochastic *SIR* model on a square lattice. The graphs compare the outbreak data obtained without any mitigation (grey) and with social distancing measures implemented for different durations $T$, as indicated. In all cases, social distancing is turned on once $I\left(t\right)$ reaches the set threshold of 10% of the total population $N.$ The resurgent outbreak is drastically reduced in both its intensity and growth rate as social distancing is maintained for longer time periods $T$. (The data for each curve were averaged over $100$ independent realizations; the shading indicates statistical error estimates.) Inset: time $\tau$ to reach the second peak following the end of the mitigation; the data indicate an exponential increase of $\tau$ with $T$.

Figure 1 depicts two sets of Monte Carlo simulation snapshots, each beginning at the moment when social distancing is switched on. The second column shows the configurations when the repulsive interaction $V$ is turned off again after respectively $T=2/a$(top), and $T=10/a$ (bottom), while the last two sets of snapshots illustrate the subsequent resurgence of the outbreak (*). With increasing mitigation duration $T$, the likelihood for the disease to locally go extinct in isolated population clusters grows markedly. As seen in the bottom row, the prevalence and spreading of the infection thus becomes confined to the perimeters of a mere few remaining centers. Hence we observe drastically improved mitigation effects for extended $T$: As shown in Fig. 2, the resurgence peak in the $I(t)$ curve assumes markedly lower values and is reached after much longer times. In fact, the time $\tau (T)$ for the infection outbreak to reach its second maximum increases exponentially with the social-distancing duration, as evidenced in the inset of Fig. 2 (see also Fig. 6 below). We emphasize that localized disease extinction and spatial confinement of the prevailing disease clusters represent correlation effects that cannot be captured in the *SIR* mean-field rate equation description.

### Two-dimensional small-world networks

In modern human societies, individuals as well as communities feature long-distance connections that represent ‘express’ routes for infectious disease spreading in addition to short-range links with their immediate neighbors. To represent this situation, we extend our regular lattice with diffusive propagation to a two-dimensional Newman-Watts small-world network^23^, which was previously applied to the study of plant disease proliferation^24^. Diffusive propagation is the manifestation of the natural movement of the individuals over the spatial extent of the lattice. In contrast to the Watts-Strogatz model^25^, in which the small-world property is generated through rewiring bonds of a one-dimensional chain of sites, a Newman-Watts small-world network may be constructed as follows: For each nearest-neighbor bond, a long-distance link (or `short-cut’) is added with probability $\varphi$ between randomly chosen pairs of vertices. As illustrated in Figure S3 (Supplemental Information), the resulting network features $2 \varphi {L}^{2}$ long-distance links, with mean coordination number $<k> = 4 (1+ \varphi )$.

Again, each vertex may be in either of the states $S$, $I$, $R$, or empty, and each individual can hop to another site along any (nearest-neighbor or long-distance) link with a total diffusion rate $d$. While network graphs have been widely employed before to represent human social interactions, we emphasize that our approach substantially differs in that we simulate a fully stochastic set of SIR reactions on dynamically changing networks that have an underlying static small-network structure. A typical snapshot of the SIR model on this small-world architecture is shown in Figure S3 (Supplemental Information). The unmitigated simulation parameters are: $L=1000, N=\mathrm{100,000}$, $I\left(0\right)=100, d=1$, and $\varphi =0.6.$ The presence of long-range links increases the mean connectivity, rendering the population more mixed, which in turn significantly facilitates epidemic outbreaks (see Figure S4 in the Supplemental Information). We remark that for the *SIR* dynamics, the Newman-Watts small-world network effectively interpolates between a regular two-dimensional lattice and a scale-free network dominated by massively connected hubs; moreover, as the hopping probability $d\to 0$, the small-world network is effectively rendered static.

**Figure 3:**
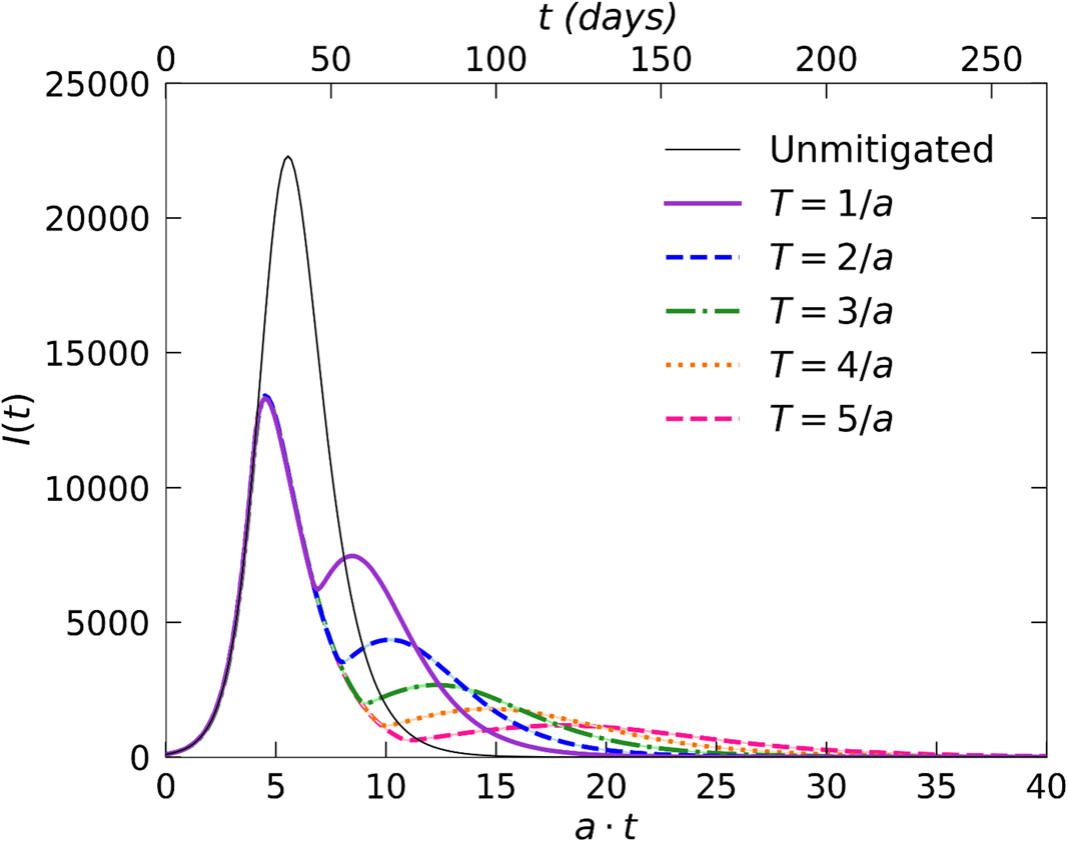
Infection curves $I(t)$ from stochastic *SIR* model simulations on a two-dimensional Newman-Watts small-world network. The graphs compare outbreak data without mitigation (grey) and for varying social-distancing intervention duration $T$(as indicated), during which the probability of moving through long-distance connections was drastically reduced to ${d}_{\varphi }=0.05$. (The data for each curve were averaged over $100$ independent realizations).

In the two-dimensional small-world network, we may introduce social-distancing measures through two distinct means: (i) We can globally diminish mobility by adopting a reduced overall diffusion rate ${d}^{^{\prime}}<1$; and/or (ii) we can drastically reduce the probability of utilizing a long-distance connection to ${d}_{\varphi }\ll 1$. We have found that the latter mitigation strategy of curtailing the infection short-cuts into distant regions has a far superior effect. Therefore, in Fig. 3 we display the resulting data for such a scenario where we set ${d}_{\varphi }=0.05$, yet kept the diffusion rate unaltered at $d=1$; as before, this control was triggered once $I\left(t\right)=0.1 N$ had been reached in the course of the epidemic. The resurgence peak height and growth rate become even more stringently reduced with extended mitigation duration than for (distinct) social distancing measures implemented on the regular lattice.

### Random and scale-free contact networks

Finally, we run the stochastic *SIR* dynamics on two different static structures, namely i) randomly connected and ii) scale-free contact networks. Each network link may be in either the $S$, $I$, or $R$ configurations, which are subject to the *SIR* reaction rules, but we do not allow movement among the network vertices. For the random network, we uniformly distribute $\mathrm{1,000,000}$ edges among $N=\mathrm{100,000}$ nodes; this yields a Poisson distribution for the connectivity with preset mean (equal to the variance) $<k> ={(\Delta k)}^{2}=20$. For the scale-free network, we employ the Barabasi-Albert graph construction^26^, where each new node is added successively with $k=4$ edges, to yield a total of $\mathrm{799,980}$ edges. The connectivity properties in these quite distinct architectures are vastly different, since the scale-free networks feature prominent `hubs’ through which many other nodes are linked. In the epidemic context, these hubs represent super-spreader centers through which a large fraction of the population may become infected^10,27^.

To implement the stochastic *SIR* dynamics on either contact network, we employ the efficient rejection-free Gillespie dynamical Monte Carlo algorithm: Each reaction occurs successively, but the corresponding time duration between subsequent events is computed from the associated probability function^28^ (for details, see Supplemental Information). The random social network may be considered an emulation of the well-connected mean-field model. Indeed, we obtain excellent agreement for the temporal evolution of the *SIR* dynamics in these two systems with $a=$ 0.15 *MCS* (for the scale-free network, a small adjustment to an effective mean-field recovery rate $a\approx$ 0.18 *MCS* is required). A variety of measures can be taken to effectively control the epidemic spread on a network^21,22^. We implement a `complete lockdown’ mitigation strategy: Once the threshold $I\left(t\right)=0.1 N$ has been reached, we immediately cut all links for a subsequent duration $T$; during that time interval, only spontaneous recovery $I \to R$ can occur.

**Figure 4:**
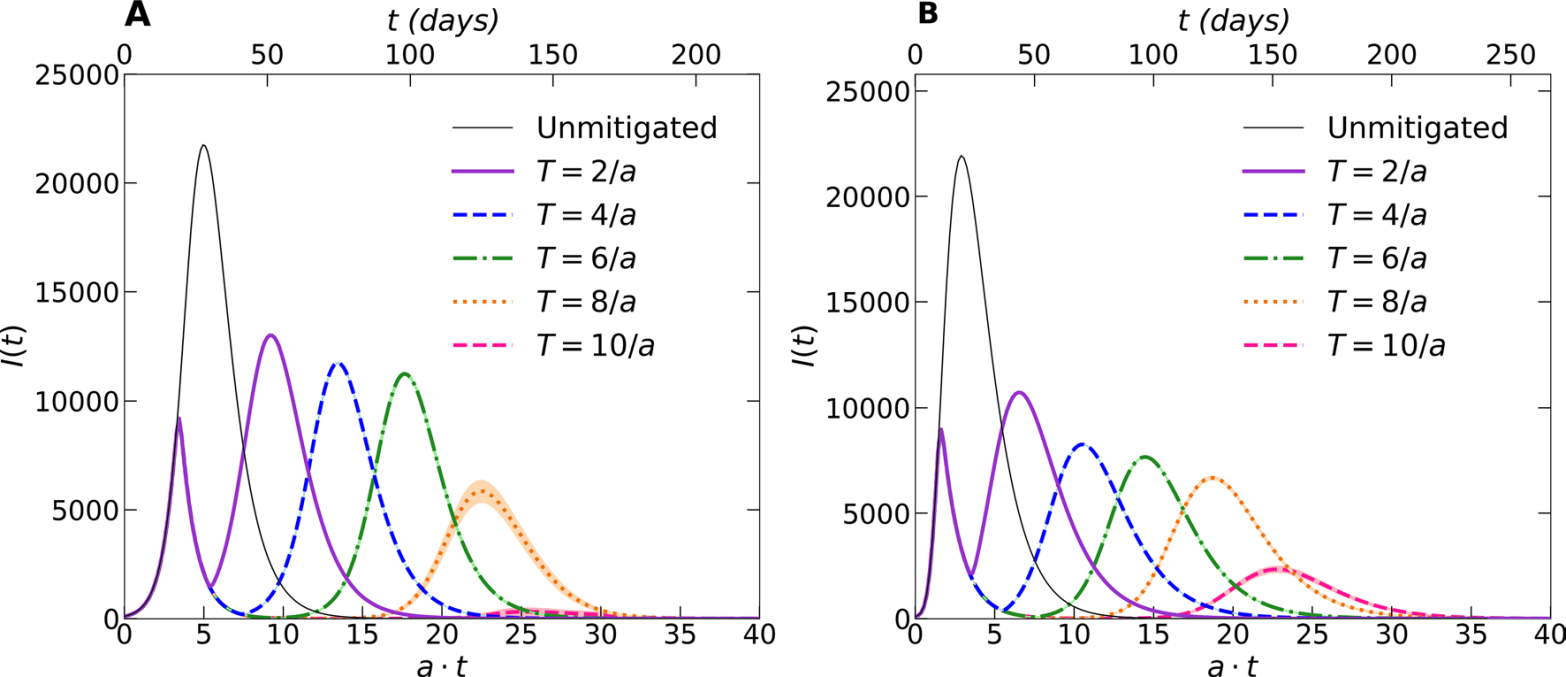
Infection outbreak curves $I(t)$ from stochastic *SIR* model simulations. (**A**) On a randomly connected network; (**B**) on a scale-free network with varying social-distancing intervention duration $T$. (The data for each curve were averaged over $100$ independent realizations).

In Fig. 4, we discern a markedly stronger impact of this lockdown on the intensity of the epidemic resurgence in both these static contact network architectures, see also Fig. 6A below. On the other hand, the mitigation duration influences the second infection wave less strongly, with the time until its peak has been reached growing only linearly with $T:$ $\tau \left(T\right) \sim T,$ as is visible in Fig. 6B. There is however a sharp descent in resurgent peak height beyond an apparent threshold $T>7/a$ for the random network, and $T>8/a$ for the scale-free network. For both the two-dimensional regular lattice and small-world structure, a similar sudden drop in the total number of infected individuals (Fig. 6B) requires a considerably longer mitigation duration: In these dynamical networks, the repopulation of nodes with infective individuals facilitates disease spreading, thereby diminishing control efficacy.

We remark that if a drastically reduced diffusivity ${d}^{^{\prime}}\ll 1$ is implemented, the small-world results closely resemble those for a randomly connected contact network (Fig. 6A).

**Figure 5:**
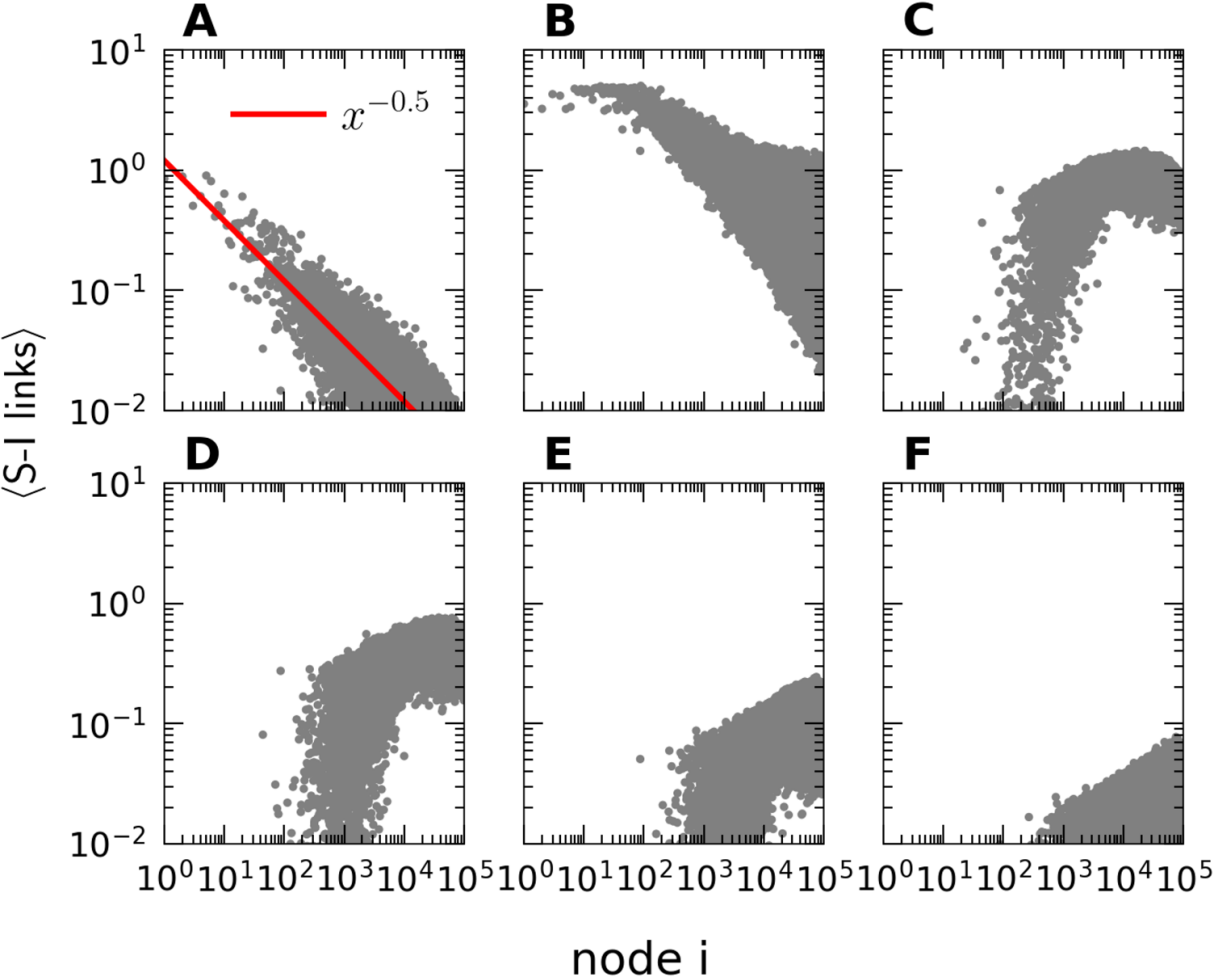
Distribution of the mean number of susceptible-infectious (*SI*) connections for the nodes in scale-free contact networks. (**A**) Network configuration before and (**B**–**F**) while the epidemic surge is spreading through the system. (**A**) at $t=0$; (**B**) after $5$ days; (**C**) after $10$ days; (**D**) after $20$ days, when the epidemic has reached its (unmitigated) peak; (**E**) after $30$ days; (**F**) after $40$ days, when only about $100$ infectious individuals are left. (The data for each curve were averaged over $\mathrm{10,000}$ independent simulation runs).

Moreover, we have observed an unexpected and drastic effective structural change in the scale-free network topology as a consequence of the epidemic outbreak infecting its susceptible nodes. Naturally, the highly connected hubs are quickly affected, and through transitioning to the recovered state, become neutralized in further spreading the disease. As shown in Fig. 5, as the infection sweeps through the network (in the absence of any lockdown mitigation), the distribution of the remaining active susceptible-infectious (*SI*) links remarkably changes from the initial scale-free power law with exponent $-1/2$ to a more uniform, almost randomized network structure. The disease resurgence wave thus encounters a very different network topology than the original outbreak.

## Discussion

**Figure 6:**
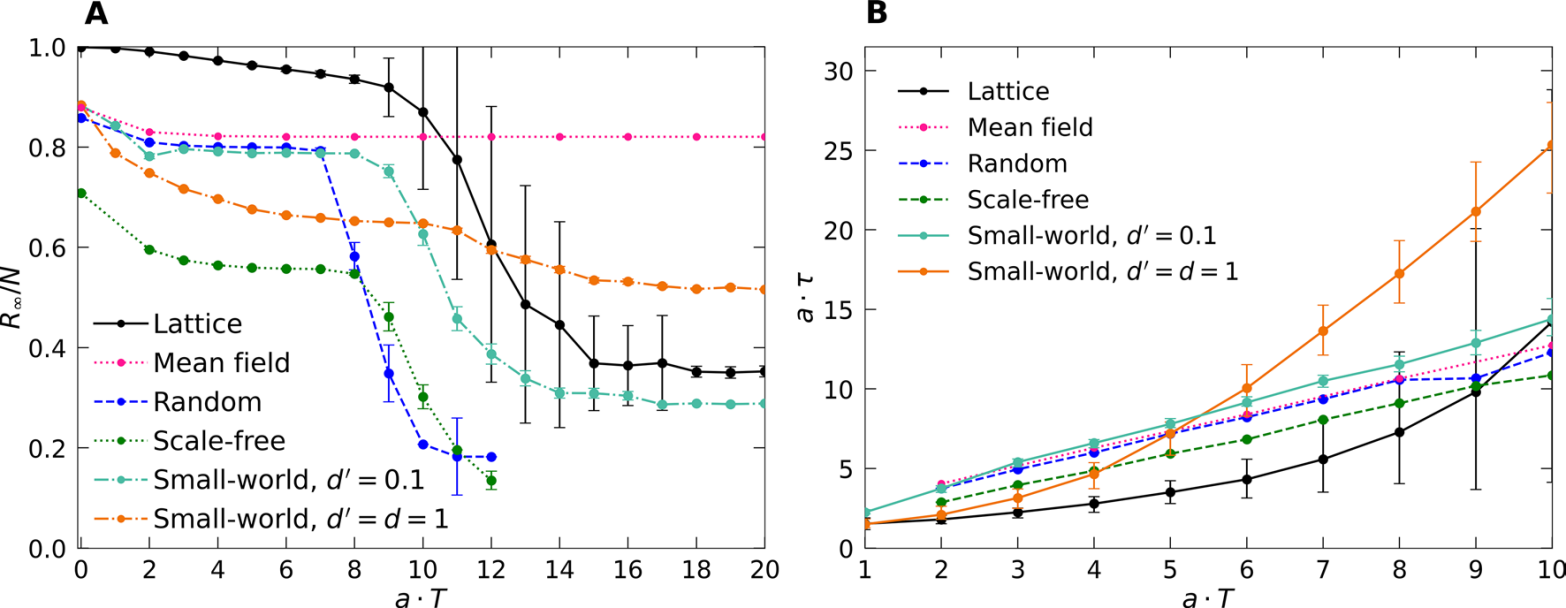
Comparison of epidemic control measures through social distancing mitigation as functions of their duration $T$ on the various architectures. (**A**) Recovered saturation fraction ${R}_{\infty }/N$; (**B**) time $\tau$ after release of the control measures until the infection resurgence peak is reached.

In this study, we implemented social distancing control measures for simple stochastic *SIR* epidemic models on regular square lattices with diffusive spreading, two-dimensional Newman-Watts small-world networks that include highly infective long-distance connections, and static contact networks, either with random connectivity or scale-free topology. In these distinct architectures, all disease spreading mitigation measures, be that through reduced mobility and/or curtailed connectivity, must of course be implemented at an early outbreak stage, but also maintained for a sufficient duration to be effective. In Fig. 6, we compare salient features of the inevitable epidemic resurgence subsequent to the elimination of social distancing restrictions, namely the asymptotic fraction ${R}_{\infty }/N$ of recovered individuals, i.e., the integrated number of infected individuals; and the time $\tau (T)$ that elapses between the release and the peak of the second infection wave, both as function of the mitigation duration $T$. We find that the latter grows exponentially with $T$ on both dynamical lattice architectures, but only linearly on the static networks (Fig. 6B). Furthermore, as one would expect, the mean-field rate equations pertaining to a fully connected system describe the randomly connected network very well.

In stark contrast to the mean-field results (indicated by the purple lines in Fig. 6), the data for the lattice and network architectures reveal marked correlation effects that emerge at sufficiently long mitigation durations $T$. For $T>8/a$ in the static networks, and $T>12/a$ in the lattice structures, the count of remaining infectious individuals $I$ becomes quite low; importantly, these are also concentrated in the vicinity of a few persisting infection centers. This leads to a steep drop in ${R}_{\infty }/N$ , the total fraction of ever infected individuals, by a factor of about $4$ in the static network, and $3$ in the dynamic lattice architectures. Thus, in these instances, follow-up disease control measures driven by high-fidelity testing and efficient contact tracking should be capable of effectively eradicating the few isolated disease resurgence centers. However, to reach these favorable configurations for the implementation of localized and targeted epidemic control, it is imperative to maintain the original social-distancing restrictions for at least a factor of three (better four) longer than it would have taken the unmitigated outbreak to reach its peak ($T\approx 3/a$ … $6/a$ in our simulations)—for COVID-19 that would correspond to about 2 months. As is evident from our results for two-dimensional small-world networks that perhaps best represent human interactions, it is also absolutely crucial to severely limit all far-ranging links between groups to less than 5% of the overall connections, during the disease outbreak.

Following this work, we have further looked at the effects of introducing the incubation period to the modeling of the epidemics spread^29^. Our simulations of an extended Susceptible-Exposed-Infectious-Recovered (SEIR) compartmental model have shown that the incubation period sets a delay to the infection onset and induces a broadening of the infection curves in comparison to the SIR model.

## Supplementary Information

Supplementary Information.

## Data Availability

All data generated or analyzed during this study are included in this published article (and its Supplementary Information files). (*) The full simulation movie files are available at: https://drive.google.com/drive/folders/14YRAlWyDbTN8L7vfwdeUMpbKlcC_fvRU?usp=sharing.

## References

[1] Brauner, J. M. *et al*. The effectiveness of eight nonpharmaceutical interventions against COVID-19 in 41 countries. MedRxiv:2020.05.28.20116102v4 (28 May 2020).

[2] Flaxman S (2020). Estimating the effects of non-pharmaceutical interventions on COVID-19 in Europe. Nature.

[3] Ferguson NM (2006). Strategies for mitigating an influenza pandemic. Nature.

[4] Halloran ME (2008). Modeling targeted layered containment of an influenza pandemic in the United States. Proc. Natl. Acad. Sci. U.S.A..

[5] Murray, C. J. L. *et al*. Forecasting COVID-19 impact on hospital bed-days, ICU-days, ventilator-days and deaths by US state in the next 4 months. MedRxiv:2020.03.27.20043752 (30 March 2020).

[6] Ferguson, N. M. *et al*. Report 9: impact of non-pharmaceutical interventions (NPIs) to reduce COVID-19 mortality and healthcare demand. 10.25561/77482 (2020). Accessed 30 March 2020. 10.1007/s11538-020-00726-x PMC7140590 32270376

[7] Adiga, A. *et al*. Evaluating the impact of international airline suspensions on the early global spread of COVID-19. MedRxiv:2020.02.20.20025882 (02 March 2020).

[8] Brockmann D, Helbing D (2013). The hidden geometry of complex, network-driven contagion phenomena. Science.

[9] Liccardo A, Fierro A (2013). A lattice model for influenza spreading. PLoS ONE.

[10] Keeling MJ, Eames KT (2005). Networks and epidemic models. J. R. Soc. Interface..

[11] Kermack WO, McKendrick AG (1927). A contribution to the mathematical theory of epidemics. Proc. R. Soc. A.

[12] Murray JD (2002). Mathematical Biology.

[13] He X (2020). Temporal dynamics in viral shedding and transmissibility of COVID-19. Nat. Med..

[14] Täuber UC (2014). Critical Dynamics—A Field Theory Approach to Equilibrium and Non-Equilibrium Scaling Behavior.

[15] Lindenberg K, Metzler R, Oshanin G (2019). Chemical Kinetics: Beyond the Textbook.

[16] Eubank S (2020). Commentary on Ferguson, et al., “Impact of non-pharmaceutical interventions (NPIs) to reduce COVID-19 mortality and healthcare demand”. Bull. Math. Biol..

[17] Wu, K. *et al*. Generalized logistic growth modeling of the COVID-19 outbreak in 29 provinces in China and in the rest of the world. arXiv:2003:05681 (12 March 2020). 10.1007/s11071-020-05862-6 PMC7437112 32836822

[18] Maharaj S, Kleczkowski A (2012). Controlling epidemic spread by social distancing: do it well or not at all. BMC Public Health.

[19] Schmittmann B, Zia RKP (1995). Statistical Mechanics of Driven Diffusive Systems (Phase Transitions and Critical Phenomena).

[20] Marro J, Dickman R (2005). Nonequilibrium Phase Transitions in Lattice Models.

[21] Gross T, Sayama H (2009). Adaptive Networks (Adaptive Networks. Understanding Complex Systems).

[22] Hindes J, Schwartz IB, Shaw LB (2018). Enhancement of large fluctuations to extinction in adaptive networks. Phys. Rev. E.

[23] Newman MEJ, Watts DJ (1999). Scaling and percolation in the small-world network model. Phys. Rev. E.

[24] Newman MEJ, Jensen I, Ziff RM (2002). Percolation and epidemics in a two-dimensional small world. Phys. Rev. E.

[25] Watts DJ, Strogatz SH (1998). Collective dynamics of 'small-world' networks. Nature.

[26] Albert R, Barabási AL (2000). Topology of evolving networks: local events and universality. Phys. Rev. Lett..

[27] Easley D, Kleinberg J (2010). Networks, Crowds, and Markets: Reasoning about a Highly Connected World.

[28] Vestergaard CL, Génois M (2015). Temporal Gillespie algorithm: fast simulation of contagion processes on time-varying networks. PLoSComput. Biol..

[29] Serrao, S. R. et al. Requirements for the containment of COVID-19 disease outbreaks through periodic testing, isolation, and quarantine. MedRxiv:2020.10.21.20217331 (25 October 2020).

