# Supplementary material for: Social distancing and epidemic resurgence in agent-based susceptible-infectious-recovered models
Source: Sci Rep. 2021 Jan 8;11:130. doi: 10.1038/s41598-020-80162-y (PMC7794373; doi:10.1038/s41598-020-80162-y)
Supplement: Supplementary file 1 — Supplementary Information. [file 41598_2020_80162_MOESM1_ESM.docx]

**Supplementary Information:**

**Social distancing and epidemic resurgence in agent-based Susceptible-Infectious-Recovered models**

Ruslan I. Mukhamadiarov^1^, Shengfeng Deng^1,2^, Shannon R. Serrao^1^, Priyanka^1^, Riya Nandi^1^, Louie Hong Yao^1^, and Uwe C. Täuber*^1,3^

^1^ Department of Physics and Center for Soft Matter and Biological Physics, Virginia Tech, Blacksburg, VA 24061, USA.

^2^ Key Laboratory of Quark and Lepton Physics (MOE) and Institute of Particle Physics, Central China Normal University, Wuhan 430079, China.

^3^ Faculty of Health Sciences, Virginia Tech, Blacksburg, VA 24061, USA.

* Uwe C. Täuber

**Email:**  [tauber@vt.edu](mailto:tauber@vt.edu)

All authors contributed equally to this work.

**Square lattices with diffusive spreading**

On our regular square lattice with $L^{2}$ sites set on a two-dimensional torus, we implement the stochastic Susceptible-Infectious-Recovered (SIR) epidemic model with the following individual-based Monte Carlo algorithm:

1. Randomly distribute $N$individuals on the lattice, subject to the restriction that each site may only contain at most one individual, and with period boundary conditions. Some small fraction of the individuals will initially be infectious, while the remainder of the population will be susceptible to the infection.
2. Perform random sequential updates $L^{2}$ times in one Monte Carlo step (*MCS*) by picking a lattice site at random, and then performing the following actions:
   1. If the selected site contains a susceptible $S$or a recovered individual $R$, a hopping direction is picked randomly. If the adjacent lattice site in the hopping direction is empty, then the chosen individual is moved to that neighboring site with hopping probability $d$ that is related to a macroscopic diffusion rate.
   2. If the chosen lattice site contains an infectious individual $I$, it will first try to infect each susceptible nearest neighbor $S$with a prescribed infection probability$r$. If this attempt is successful, the involved susceptible neighbor $S$immediately changes its state to infected $I$. After the originally selected infected individual has repeated its infection attempts with all neighboring susceptibles $S$, it may reach the immune state $R$with recovery probability $a$. Finally, this particular individual, whether still infectious or recovered, tries to hop in a randomly picked direction with probability $d$, provided the chosen adjacent lattice site is empty.
3. Repeat the procedures in item 2 for a preselected total number of Monte Carlo steps.

*Figure S1 about here.*

To determine the effective (coarse-grained) basic epidemic reproduction ratio $R_{0}$, we fit the infection curves to straightforward numerical integrations of the deterministic *SIR* rate equations $dS(t)/dt= -r S\left( t \right) I\left( t \right)/N$, $dI(t)/dt= r S\left( t \right) I\left( t \right)/N-a I\left( t \right)$, $dR(t)/dt= a I\left( t \right)$, and adjust the lattice simulation infection probability $r\approx1.0$ and to a lesser extent, the recovery probability $a$ to finally match the targeted COVID-19 value $R_{0}\approx2.4$. We note that this slightly ‘renormalized’ value for $a$ is subsequently utilized to set the time axis scale in the figures. On the mean-field level, initially $R_{0}=(r/a) S(0)/N$, since all nodes are mutually connected. In spatial settings, $S\left( 0 \right)/N$is to be replaced with the mean connectivity (i.e., the coordination number for a regular lattice) to susceptible individuals. The lattice simulation data is fitted with the mean-field result by matching two parameters: the maximum value and the half-peak width of the infectious population curve $I\left( t \right)$, see Figure S1. The lattice simulation curve digresses from the mean-field curves at low $I(t)$ values, far away from the peak region. In the lattice simulations, the initial rise of the infectious population curve exhibits power-law growths $I\left( t \right) \sim t^{1.4\pm0.1}$ and $R\left( t \right) \sim t^{2.3\pm0.1}$ in clear contrast with the simple exponential rise of the mean-field *SIR* curve as obtained from integrating the mean-field rate equations. We note that these are the standard critical exponents $\theta$ and $1+\theta$ for the temporal growth of an active seed cluster near a continuous non-equilibrium phase transition to an absorbing extinction state *(11)*.

*Figures S2 about here.*

Figure S2 shows the dependence of the asymptotic number of recovered individuals $R_{\infty}$ on the density $\rho$ for various sets of hopping rates $d$ and initial infectious population values $I(0)$. These data indicate the existence of a well-defined epidemic threshold, i.e., a percolation-like sharp transition from a state when only a tiny fraction of individuals is infected, to the epidemic state wherein the infection spreads over the entire population *(11)*. As one would expect, this critical point depends only on the ratio $a/d$ of the recovery and hopping rates. Varying the lattice simulation parameters just shifts the location of the epidemic threshold. Once the model parameters are set in the epidemic spreading regime, the system’s qualitative behavior is thus generic and robust, and only weakly depends on precise parameter settings.

**Two-dimensional small-world networks**

*Figure S3 about here.*

For our two-dimensional small-world network, whose construction is schematically depicted in Figure S3, we employ a similar Monte Carlo algorithm as described above; the essential difference is that individuals may now move to adjacent nearest-neighbor as well as to distant lattice sites along the pre-set ‘short-cut’ links. Figure S4A demonstrates (for fixed diffusivity $d=1$) that as function of the fraction $\varphi$ of long-distance links in a two-dimensional small-world network, the epidemic threshold resides quite close to zero: The presence of a mere few ‘short-cuts’ in the lattice already implies a substantial population mixing. The inset, where the $\varphi$ axis is scaled logarithmically, indicates that sizeable outbreaks begin for $\varphi\geq0.05$. Figure S4B similarly shows the outbreak dependence on the diffusion rate $d$ (here for $\varphi=0.6$), with the threshold for epidemic spreading observed at $d\approx0.3$. Evidently, prevention of disease outbreaks in this architecture requires that both mobility and the presence of far-ranging connections be stringently curtailed.

*Figure S4 about here.*

**Random and scale-free contact networks**

For both the randomly connected and scale-free contact networks, we employ the Gillespie or dynamical Monte Carlo algorithm, which allows for efficient numerical simulations of Markovian stochastic processes. It consists of these subsequent steps:

1. Initially, few nodes are assigned to be infected $I$, while all other nodes are set in the susceptible state $S$. Each susceptible node $S$ is characterized by a certain number of active links that are connected to infected nodes $I$.
2. We then determine the rate at which each infected node $I$ will recover, and at which each susceptible node $S$ with a non-zero number of active links becomes infected. From these we infer the total event rate $r_{tot}$.
3. Based on this total rate $r_{tot}$, we select the waiting time until the next event occurs from an exponential distribution with mean $r_{tot}$.
4. We then select any permissible event with a probability proportional to its rate, update the status of each node, and repeat these processes for the desired total number of iterations.

*
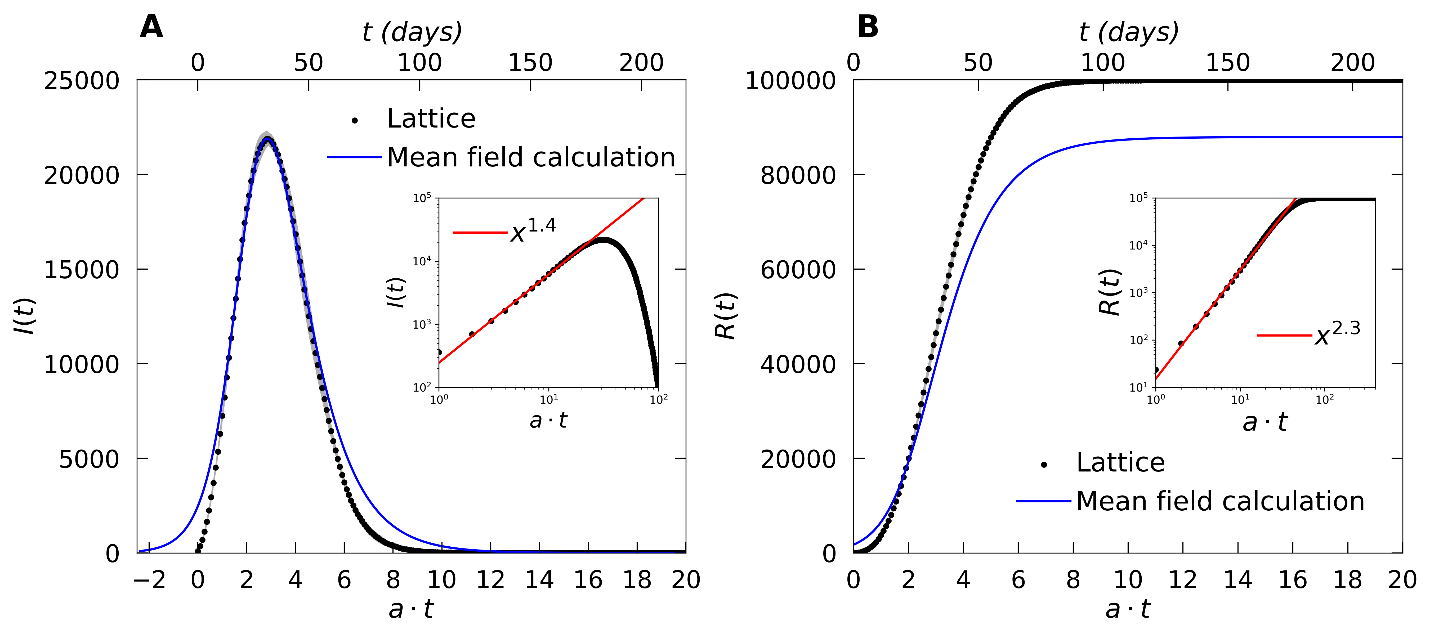
*

Figure S1. **Fit of infection curves from lattice simulations to the numerically integrated curves from the mean-field *SIR* rate equations.** (**A**) Infectious population $I\left( t \right)$; (**B**) recovered number of individuals $R(t)$. The insets illustrate the power law initial growth $I\left( t \right) \sim t^{1.4\pm0.1}$ and $R\left( t \right) \sim t^{2.3\pm0.1}$ for the lattice simulation data (averaged over $100$independent realizations).

*
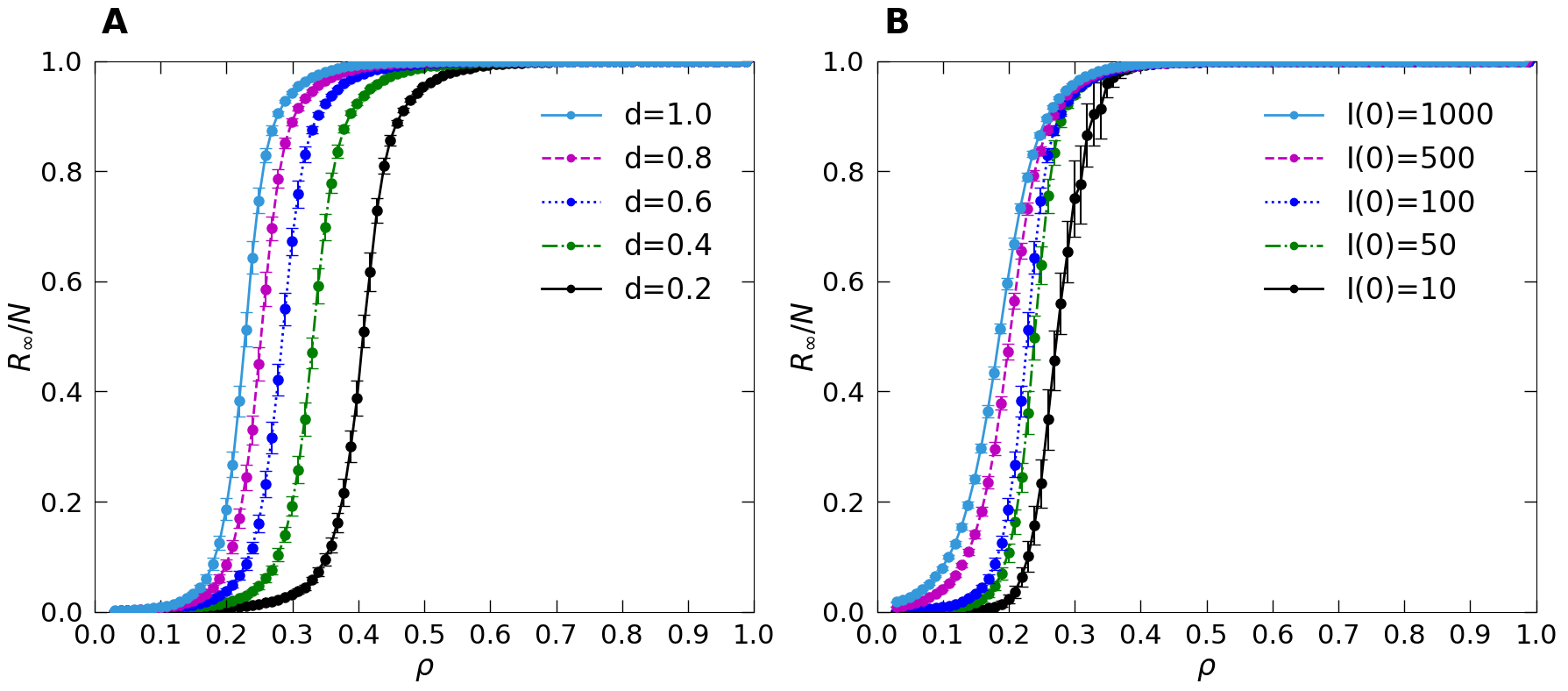
*

Figure S2: **Variation of the total fraction of recovered individuals** $\boldsymbol{R}_{\boldsymbol{\infty}}\boldsymbol{/N}$ **with the lattice simulation parameters in stochastic *SIR* model simulations on a square lattice on the total density** $\boldsymbol{\rho}$. (**A**) Data for different sets of nearest-neighbor hopping rates $d$; (**B**) for varying numbers of initially infected individuals $I(0)$. The graphs demonstrate the presence of a percolation-like epidemic threshold. (Each data point was averaged over $100$independent simulation runs.)


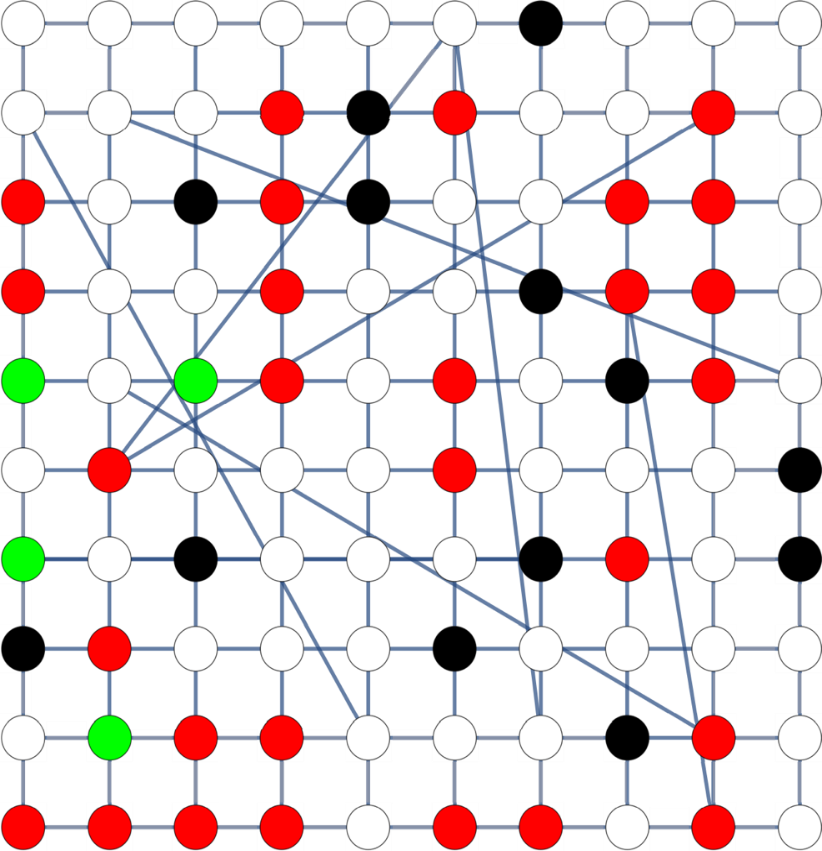


Figure S3: **Schematic construction of a two-dimensional Newman-Watts small-world network.** It is obtained from a regular square lattice through adding long-distance connections; shown is an *SIR* model configuration snapshot with empty, susceptible, infectious, and recovered states.

**
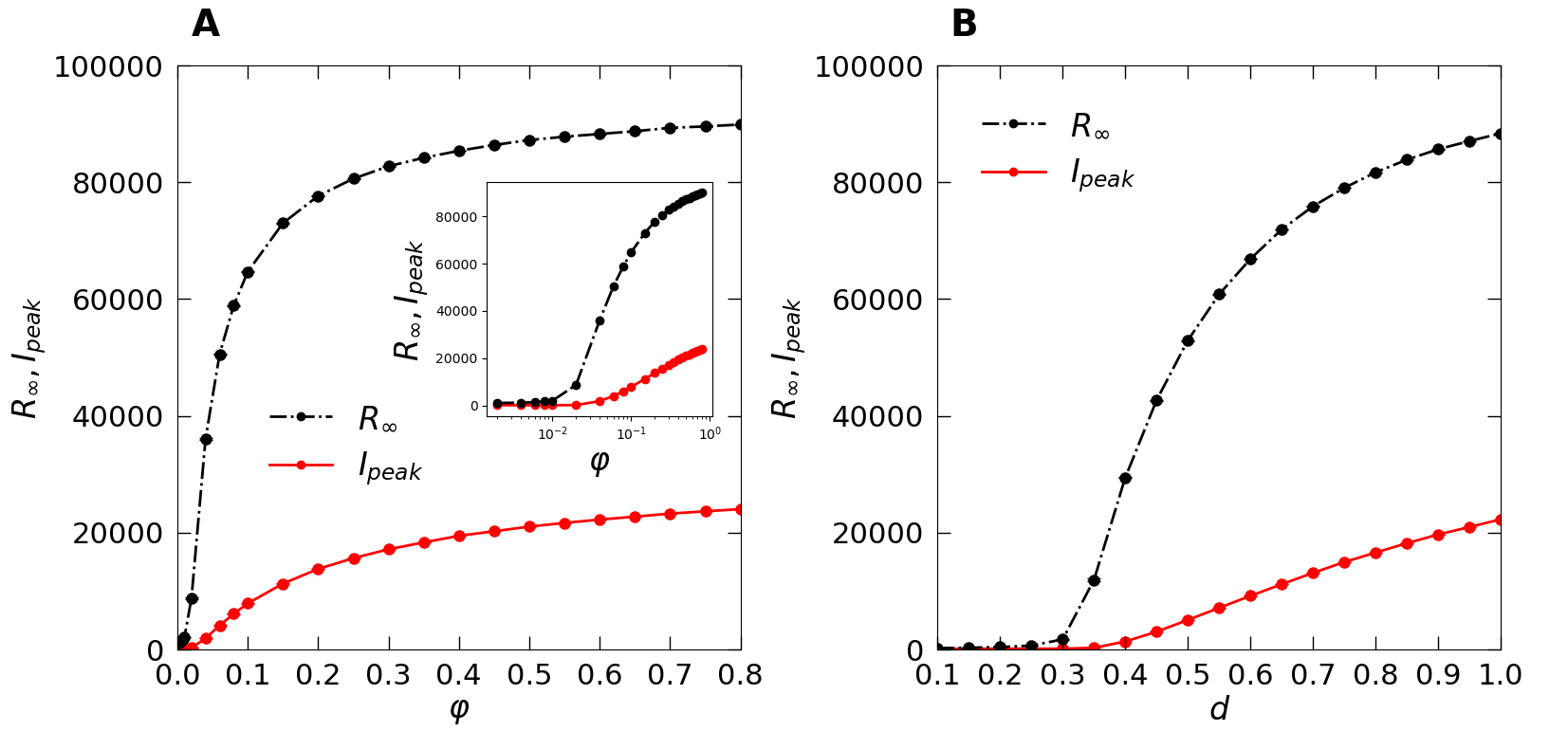
**

Figure S4: **Epidemic peak value and total recovered fraction** $\boldsymbol{R}_{\boldsymbol{\infty}}$**and peak intensity** $\boldsymbol{I}_{\boldsymbol{peak}}$**.** (**A**) As functions of the fraction $\varphi$ of long-distance links with fixed $d=1$ (inset: same data, with the $\varphi$ axis on a logarithmic scale); (**B**) as functions of the diffusivity $d$ with fixed $\varphi=0.6$for the SIR model implemented on a two-dimensional Newman-Watts small-world network. (Each data point was averaged over $100$independent realizations.)
